# Supplementary material for: Single protein encapsulated SN38 for tumor-targeting treatment
Source: J Transl Med. 2023 Dec 10;21:897. doi: 10.1186/s12967-023-04778-0 (PMC10712105; doi:10.1186/s12967-023-04778-0)
Supplement: Supplementary file 1 — Additional file 1: Fig. S1. Tumor volume of different treatment groups on Day 10. SPESN38-5 by IV was more efficacious than clinical drug irinotecan, while PO route was not effective. Fig. S2. Tumor volume comparison on Day 21 (ending day of experiments). SPESN38-5 by IV was significantly efficacious than irinotecan. Fig. S3. Tumor weigh comparison of different treatment groups on ending days. The mice from the control and PO groups had to been sacrificed on Day 10 due to the large tumor size. Table S1. Summary of antitumor efficacy of irinotecan and SPESN38-5 Against HCT-116. Table S2. Summary of antitumor efficacy of DOX and SPESN38-8 against SK-LMS-1. Table S3. Summary of PK parameters for irinotecan at 200 mg/kg from literature. Table S4. Summary of tumor volume change for all groups on Day 10. Table S5. Summary of tumor volume change for all groups on Day 21. Table S6. Summary of tumor wight for all groups on the ending days. Table S7. Summary of tumor volume by day and group. Table S8. Summary of tumor volume change from Day 0 by different groups and days. Table S9. Result summary of Tukey-Kramer adjusted pairwise tests. Table S10. Tumor weight summary on their ending days [file 12967_2023_4778_MOESM1_ESM.docx]

**Supplementary Information**

**Single Protein Encapsulated SN38 for Tumor-Targeting Treatment**

Changjun Yu^1,2*^, Faqing Huang^3*^, Kinsley Wang^2^, Mengmeng Liu^2^, Warren A. Chow^4^,

Xiang Ling^5,6^, Fengzhi Li^5^, Jason L. Causey^7^, Xiuzhen Huang^8^, Galen Cook-Wiens^9^ and Xiaojiang Cui^10*^

Author Affiliations:

^1^ Department of Chemistry, California Institute of technology, Pasadena, CA 91125, USA

^2^ Sunstate Biosciences, LLC, 870 S. Myrtle Ave, Monrovia, CA 91016. USA

^3^ Department of Chemistry and Biochemistry, University of Southern Mississippi, Hattiesburg, MS 39406, USA

^4^ Division of Hematology/Oncology, Department of Medicine, UCI Health, Orange, CA 92868, USA

^5^ Department of Pharmacology & Therapeutics, Roswell Park Comprehensive Cancer Center, Elm and Carlton Streets, Buffalo, NY 14263, USA

^6^ Canget BioTekpharma, LLC, 701 Ellicott Street, Buffalo, NY 14203, USA

^7^ Department of Computer Sciences, Arkansas State University, Jonesboro, AR 72467, USA

^8^ Department of Computational Medicine, Cedars-Sinai Medical Center, Los Angeles, CA 90048, USA

^9^ Department of Biomedical Sciences, Cedars Sinai Medical Center, Los Angeles, CA 90048, USA

^10^ Department of Surgery, Samuel Oschin Comprehensive Cancer Institute, Cedars Sinai Medical Center, Los Angeles, CA 90048, USA

**Comparison of Tumor Growth Inhibition and Statistical Analysis**

*In vivo efficacy evaluation of SPESN38-5 on HCT-116*.

Tumor volumes of different treatment groups on Day 10 were presented as box plots, showing significant tumor inhibition by SPESN38-5 via the IV route but not the PO route (**Fig. S1**). It is obvious that SPESN38-5 had better efficacy than the clinical drug irinotecan. Since there was a significant difference (p < 0.001), a Dunnett’s post-hoc analysis was performed, comparing several treatments with the control at 95% family-wise confidence level. The p-values were calculated and are shown in **Table S4**. On Day 21 when the in vivo study was completed, box plots clearly show superior efficacy of SPESN38-5 over irinotecan (**Fig. S2**). Dunnett’s post-hoc analysis generated p-values are presented in **Table S5**. Additionally, analysis of tumor weights on the ending day of the treatment are presented in box plots (**Fig. S3**). The data confirm that SPESN38-5 is much better than irinotecan at inhibiting tumor growth. Dunnett’s post-hoc analysis generated p-values at 95% family-wise confidence level are presented in **Table S6**.

In vivo efficacy evaluation of SPESN38-8 on SK-LMS-1

Tumor volume, summary of tumor volume by different days and groups are shown in **Table S7**. Summary of tumor volume change from Day 0 (volume minus day 0 volume) by different groups and days are presented in **Table S8**.

A mixed linear regression model was made with tumor volume as the outcome on day 2, 6, and 9. The model tested for group differences adjusted for time, day 0 volume, and sex. There was a within animal correlation modeled using a random intercept. DOX and SPESN38-8 groups had significantly different tumor volume on day 0, but neither was significantly different from vehicle on day 0. A significant interaction between group and time was found (type III F test p<0.0001) along with significant group differences (the group differences varied depending on day). Sex differences were not significant (type III F test p=0.9124). There was a significant difference in average tumor volume at starting on day 2 between SPESN38-8 and vehicle (p=0.0212). On day 9 all three group comparisons were significant after adjustment for multiplicity (p<0.0001 for all). **Table S9** shows results from Tukey-Kramer adjusted pairwise tests between groups at each day. The black color columns are within group at between days, the red columns are between group at each day.

Furthermore, tumor weight, summary of tumor weight in three groups for vehicle, DOX, and SPESN38-8 at their ending days are shown in **Table S10**. The ANOVA for tumor weight indicated some significant difference among the groups (*p* <0.0001). The Tukey-Kramer adjusted *p*-values between groups all indicated significant differences in average tumor weight between DOX and SPESN38-8 (adjusted *p* =0.0002, ***), between DOX and vehicle (adjusted *p* = 0.0005, ***) and between SPESN38-8 and vehicle (adjusted *p* <0.0001, ***).

*
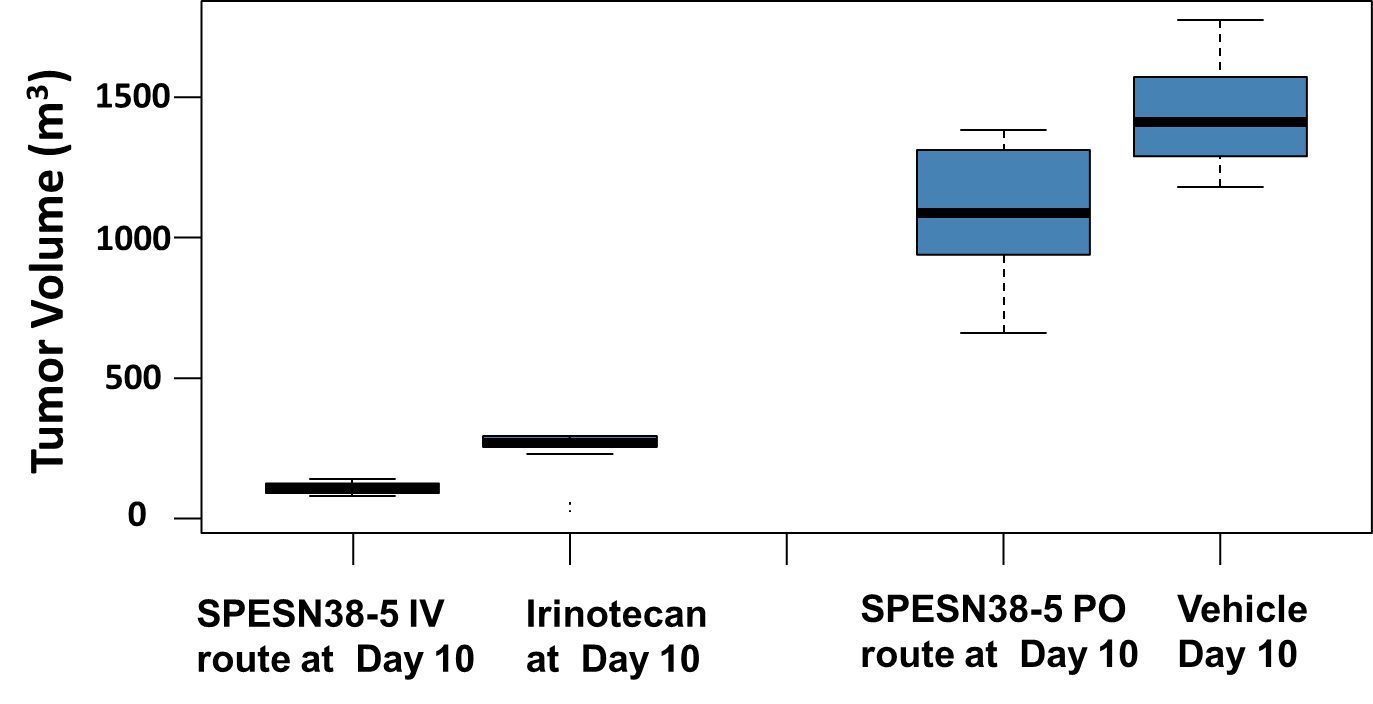
*

**Fig. S1.** Tumor volume of different treatment groups on Day 10. SPESN38-5 by IV was more efficacious than clinical drug irinotecan, while PO route was not effective.


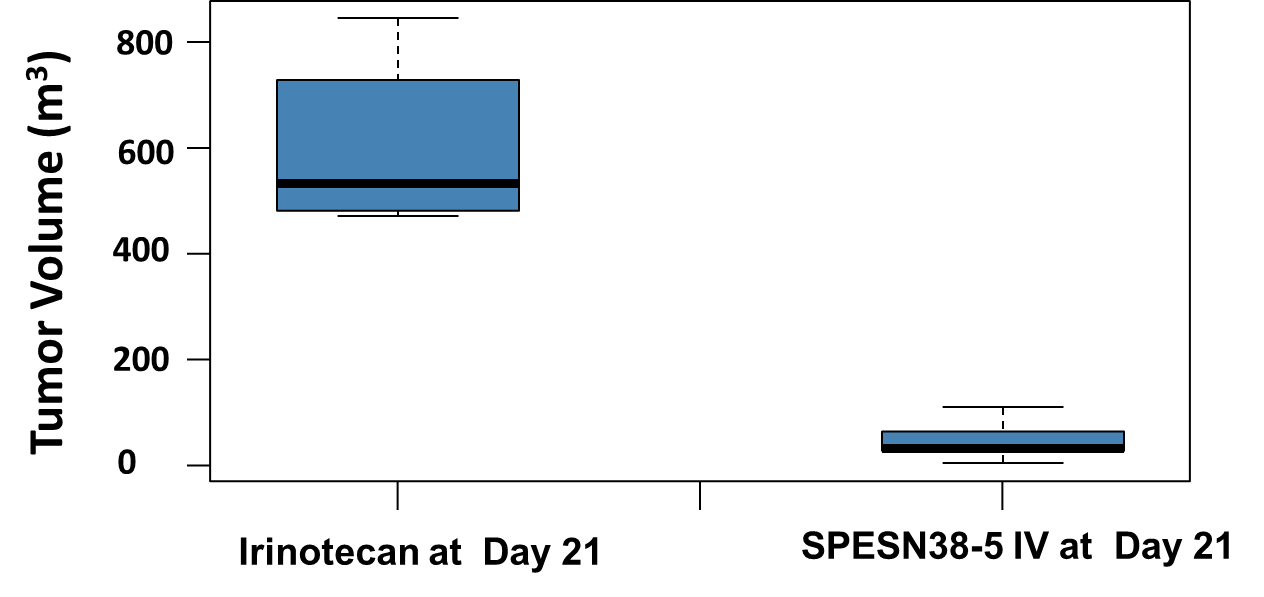


**Fig. S2.** Tumor volume comparison on Day 21 (ending day of experiments). SPESN38-5 by IV was significantly efficacious than irinotecan.

*
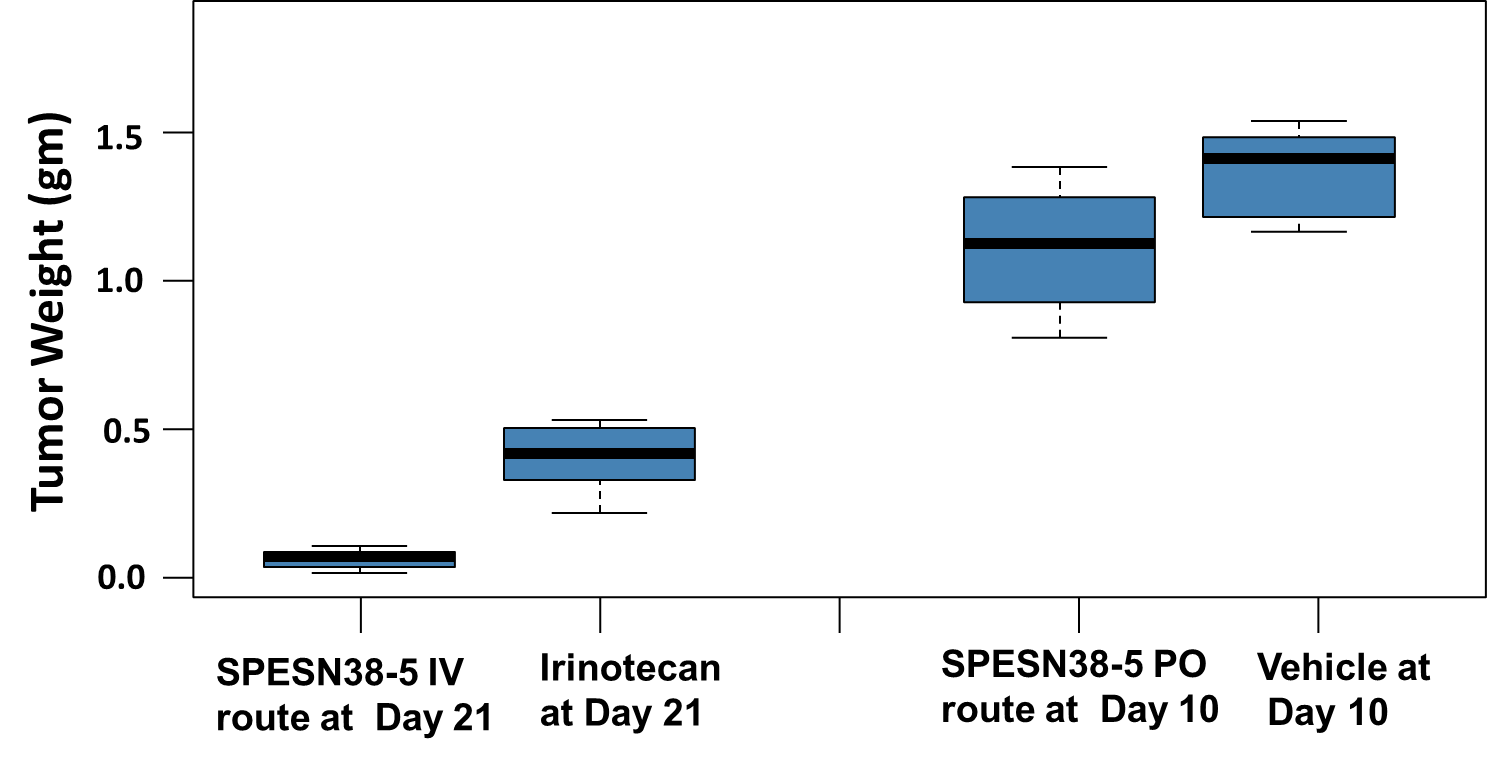
*

**Fig. S3***.* Tumor weigh comparison of different treatment groups on ending days. The mice from the control and PO groups had to been sacrificed on Day 10 due to the large tumor size.

| **Table S1**. Summary of antitumor efficacy of irinotecan and SPESN38-5 Against HCT-116 | | | | |
| --- | --- | --- | --- | --- |
|  | Control group | SPESN38-5 PO  (200 mg/kg, qwk x 2) | Irinotecan group  (50 mg/kg. qwk x 3) | SPESN38-5 IV  (55 mg/kg. qwk x 3) |
| Cumulative dose (mg/kg) | 0 | 400 | 150 | 165 |
| Ending day | 10 | 10 | 21 | 21 |
| Initial mean Tumor volume (mm^3^) | 196.2 | 186.9 | 201.6 | 182.1 |
| Mean Tumor volume (mm^3^) at Day 10 | 1435.9 | 1089.8 | 253.8 | 48.8 |
| % Tumor volume change at day 10 | 631.8 % | 483.1 % | 20.6% | -73.2 % |
| Mean Tumor volume (mm^3^) at Day 21 | N/A | N/A | 597.8 | 47.2 |
| % Tumor volume change from Day 10 to Day 21 | N/A | N/A | 135.5% | -3.3 % |
| % Tumor volume change at Day 0 to Day 21 | N/A | N/A | 196.5% | -74.1 % |
| Mean % BW change at ending day | 96.2 | 101.4 | 90.5 | 95.3 |

| **Table S2.** Summary of antitumor efficacy of DOX and SPESN38-8 against SK-LMS-1 | | | |
| --- | --- | --- | --- |
|  | Control group | DOX group (5 mg/kg, qwk x 2) | SPESN38-8 IV (33 mg/kg. qwk x 3) |
| Cumulative dose (mg/kg) | 0 | 10 | 99 |
| Ending day | 9 | 9 | 21 |
| Initial mean Tumor volume (mm^3^) | 296.5 | 334.2 | 260.1 |
| Mean Tumor volume (mm^3^) at Day 9 | 1129.5 | 843.5 | 155.8 |
| % Tumor volume change at Day 9 | 280.9 % | 142.4 % | -40.1% |
| Mean Tumor volume (mm^3^) at Day 21 | N/A | N/A | 9.5 |
| % Tumor volume change from Day 9 to Day 21 | N/A | N/A | -93.9% |
| % Tumor volume change at Day 0 to Day 21 | N/A | N/A | -96.3% |
| Mean % BW change at ending day | 100.1 | 75.4 | 100.4 |

| **Table S3**. Summary of PK parameters for irinotecan at 200 mg/kg from literature | | | | |
| --- | --- | --- | --- | --- |
| Moieties | C_max_ (ng/mL) | T_max_ (h) | T_1/2_ (h) | AUC_0-∞_ (ng x h/mL) |
| Irinotecan | 6,571 | 0.29 | 2.3 | 16,348 |
| Free SN38 | 1,125 | 0.53 | 6.0 | 9,940 |
| SN38-G | 2,620 | 0.6 | 6.6 | 15,493 |

| **Table S4**. Summary of tumor volume change for all groups on Day 10 | | | | |
| --- | --- | --- | --- | --- |
| Comparison Pairs | Difference | Lwr.ci | Upr.ci | *P* value |
| SPESN38-5 PO route-Vehicle | -346.1592 | -541.886 | -150.4325 | 0.00022 *** |
| Irinotecan-Vehicle | -1182.2163 | -1393.625 | -970.8072 | < 2e-16 *** |
| SPESN38-5 IV-Vehicle | -1387.1567 | -1582.884 | -1191.4300 | < 2e-16 *** |
| Irinotecan-SPESN38-5 PO | -836.0571 | -1018.609 | -653.5050 | 1.7e-14 *** |
| SPESN38-5 IV-SPESN38-5 PO | -1040.9975 | -1210.008 | -871.9871 | < 2e-16 *** |
| SPESN38-5 IV-Irinotecan | -204.9404 | -385.9597 | -23.92121 | 0.0241 * |

| **Table S5**. Summary of tumor volume change for all groups on Day 21 | | | | |
| --- | --- | --- | --- | --- |
| Comparison Pairs | Difference | Lwr.ci | Upr.ci | *P* value |
| SPESN38-5 IV-Irinotecan | -550.582 | -757.8372 | -343.32678 | 1.6e-05 *** |

| **Table S6**. Summary of tumor wight for all groups on the ending days | | | | |
| --- | --- | --- | --- | --- |
| Comparison Pairs | Difference | Lwr.ci | Upr.ci | *P* value |
| SPESN38-5 PO on Day 10-Vehicle on Day 10 | -0.3018750 | -0.527486 | -0.0762645 | 0.0058 ** |
| Irinotecan at Day 21-Vehicle on Day 10 | -0.960542 | -1.204229 | -0.7168544 | 2.9e-13*** |
| SPESN38-5 IV on Day 21-Vehicle on Day 10 | -1.3309464 | -1.564476 | -1.0974173 | < 2e-16 *** |
| Irinotecan on Day 21-SPESN38-5 PO on Day 10 | -0.6586667 | -0.863749 | -0.4535847 | 2.0e-08 *** |
| SPESN38-5 IV on Day 21-SPESN38-5 PO on Day 10 | -1.0290714 | -1.225604 | -0.8325383 | 2.2e-14 *** |
| SPESN38-5 IV on Day 21-Irinotecan on Day 21 | -0.3704048 | -0.523339 | -0.2174699 | 4.6e-05 *** |

| **Table S7.** Summary of tumor volume by day and group | | | | | | | |
| --- | --- | --- | --- | --- | --- | --- | --- |
| Day | Group | N | Mean | Std Dev | Minimum | Median | Maximum |
| 0 | DOX | 8 | 334.20 | 34.01 | 281.75 | 343.40 | 371.25 |
|  | SPESN38-8 | 8 | 260.11 | 44.81 | 186.23 | 260.65 | 329.23 |
|  | Vehicle | 8 | 296.46 | 36.35 | 244.09 | 305.38 | 335.23 |
| 2 | DOX | 8 | 347.68 | 68.42 | 220.34 | 355.78 | 421.65 |
|  | SPESN38-8 | 8 | 155.97 | 54.04 | 74.11 | 165.22 | 219.01 |
|  | Vehicle | 8 | 450.43 | 78.75 | 328.30 | 463.34 | 534.65 |
| 6 | DOX | 8 | 495.42 | 183.15 | 305.61 | 464.84 | 859.95 |
|  | SPESN38-8 | 8 | 213.72 | 106.53 | 135.66 | 185.73 | 452.47 |
|  | Vehicle | 8 | 678.13 | 126.33 | 506.25 | 663.20 | 850.00 |
| 9 | DOX | 8 | 843.52 | 177.65 | 617.86 | 810.34 | 1103.87 |
|  | SPESN38-8 | 8 | 155.86 | 49.92 | 70.76 | 150.23 | 226.80 |
|  | Vehicle | 8 | 1129.50 | 253.46 | 661.09 | 1180.24 | 1385.97 |
| 13 | SPESN38-8 | 8 | 74.34 | 44.67 | 44.80 | 64.85 | 182.27 |
| 16 | SPESN38-8 | 7 | 32.22 | 14.02 | 15.25 | 28.80 | 47.40 |
| 20 | SPESN38-8 | 4 | 15.93 | 13.27 | 0.00 | 18.00 | 27.74 |
| 21 | SPESN38-8 | 7 | 9.50 | 15.05 | 0.00 | 0.00 | 35.94 |

| **Table S8.** Summary of tumor volume change from Day 0 by different groups and days | | | | | | | | |
| --- | --- | --- | --- | --- | --- | --- | --- | --- |
| Day | Group | N Obs | N | Mean | Std Dev | Minimum | Median | Maximum |
| 2 | DOX | 8 | 8 | 13.48 | 50.37 | -66.31 | 14.71 | 83.90 |
|  | SPESN38-8 | 8 | 8 | -104.14 | 49.14 | -172.65 | -97.59 | -34.40 |
|  | Vehicle | 8 | 8 | 153.96 | 65.88 | 37.88 | 185.65 | 208.38 |
| 6 | DOX | 8 | 8 | 161.22 | 169.25 | -29.95 | 138.34 | 504.21 |
|  | SPESN38-8 | 8 | 8 | -46.39 | 97.60 | -127.32 | -78.34 | 175.87 |
|  | Vehicle | 8 | 8 | 381.67 | 99.76 | 248.20 | 375.98 | 514.77 |
| 9 | DOX | 8 | 8 | 509.32 | 151.91 | 331.21 | 472.65 | 748.14 |
|  | SPESN38-8 | 8 | 8 | -104.25 | 43.92 | -154.12 | -110.88 | -49.81 |
|  | Vehicle | 8 | 8 | 833.04 | 224.24 | 416.99 | 877.98 | 1050.74 |
| 13 | SPESN38-8 | 8 | 8 | -185.77 | 52.22 | -262.44 | -190.49 | -94.33 |
| 16 | SPESN38-8 | 8 | 7 | -221.72 | 34.89 | -285.23 | -210.60 | -170.68 |
| 20 | SPESN38-8 | 8 | 4 | -235.04 | 60.45 | -303.29 | -239.20 | -158.49 |
| 21 | SPESN38-8 | 8 | 7 | -244.45 | 38.31 | -303.18 | -234.90 | -186.23 |

| **Table S9.** Result summary of Tukey-Kramer adjusted pairwise tests | | | | | | | | | | |
| --- | --- | --- | --- | --- | --- | --- | --- | --- | --- | --- |
| Group | Day | Group | Day | Estimate | Standard Error | DF | t Value | Pr > \|t\| | Adjustment | Adj *P* |
| DOX | 2 | SPESN38-8 | 2 | 42.5383 | 68.8077 | 43.4 | 0.62 | 0.5397 | Tukey-Kramer | 0.9994 |
| DOX | 2 | Vehicle | 2 | -178.73 | 61.6139 | 51.7 | -2.90 | 0.0055 | Tukey-Kramer | 0.1176 |
| DOX5 | 2 | DOX | 6 | -147.74 | 52.9080 | 42 | -2.79 | 0.0078 | Tukey-Kramer | 0.1482 |
| DOX5 | 2 | DOX | 9 | -495.84 | 52.9080 | 42 | -9.37 | <.0001 | Tukey-Kramer | <.0001 |
| SPESN38-8 | 2 | Vehicle | 2 | -221.27 | 61.4210 | 51.9 | -3.60 | 0.0007 | Tukey-Kramer | 0.0212 * |
| SPESN38-8 | 2 | SPESN38-8 | 6 | -57.7508 | 52.9080 | 42 | -1.09 | 0.2813 | Tukey-Kramer | 0.9723 |
| SPESN38-8 | 2 | SPESN38-8 | 9 | 0.1078 | 52.9080 | 42 | 0.00 | 0.9984 | Tukey-Kramer | 1.0000 |
| Vehicle | 2 | Vehicle | 6 | -227.71 | 52.9080 | 42 | -4.30 | <.0001 | Tukey-Kramer | 0.0029 |
| Vehicle | 2 | Vehicle | 9 | -679.08 | 52.9080 | 42 | -12.84 | <.0001 | Tukey-Kramer | <.0001 |
| DOX | 6 | SPESN38-8 | 6 | 132.53 | 68.8077 | 43.4 | 1.93 | 0.0607 | Tukey-Kramer | 0.6004 |
| DOX | 6 | Vehicle | 6 | -258.70 | 61.6139 | 51.7 | -4.20 | 0.0001 | Tukey-Kramer | 0.0039** |
| DOX | 6 | DOX5 | 9 | -348.11 | 52.9080 | 42 | -6.58 | <.0001 | Tukey-Kramer | <.0001 |
| SPESN38-8 | 6 | Vehicle | 6 | -391.22 | 61.4210 | 51.9 | -6.37 | <.0001 | Tukey-Kramer | <.0001*** |
| SPESN38-8 | 6 | SPESN38-8 | 9 | 57.8586 | 52.9080 | 42 | 1.09 | 0.2804 | Tukey-Kramer | 0.9720 |
| Vehicle | 6 | Vehicle | 9 | -451.37 | 52.9080 | 42 | -8.53 | <.0001 | Tukey-Kramer | <.0001 |
| DOX | 9 | SPESN38-8 | 9 | 538.49 | 68.8077 | 43.4 | 7.83 | <.0001 | Tukey-Kramer | <.0001*** |
| DOX | 9 | Vehicle | 9 | -361.96 | 61.6139 | 51.7 | -5.87 | <.0001 | Tukey-Kramer | <.0001*** |
| SPESN38-8 | 9 | Vehicle | 9 | -900.45 | 61.4210 | 51.9 | -14.66 | <.0001 | Tukey-Kramer | <.0001*** |

| **Table S10.** Tumor weight summary on their ending days | | | | | | | |
| --- | --- | --- | --- | --- | --- | --- | --- |
| Group | N Obs | N | Mean | Std Dev | Minimum | Median | Maximum |
| DOX | 8 | 8 | 0.73 | 0.27 | 0.28 | 0.71 | 1.20 |
| SPESN38-8 | 7 | 7 | 0.01 | 0.01 | 0.00 | 0.00 | 0.03 |
| Vehicle | 8 | 8 | 1.37 | 0.39 | 0.80 | 1.29 | 1.90 |
